# Chinese Personality Traits and Mental Health: A Meta-Analysis

**DOI:** 10.3390/bs13080683

**Published:** 2023-08-16

**Authors:** Liangsheng Wang, Yong Zhang, Zhiyuan Fu

**Affiliations:** School of Education and Psychology, Southwest Minzu University, Chengdu 610225, China

**Keywords:** Chinese personality, mental health, psychoticism, extraversion, neuroticism

## Abstract

This meta-analysis aimed to synthesize the evidence on the relationship between Chinese personality traits and mental health. Through literature search and screening, a total of 70 original articles and 72 independent samples with a total of 65,133 participants were included. The results showed that: (1) Chinese mental health was significantly correlated with three dimensions of personality: psychoticism, neuroticism, and extraversion (correlation coefficients were 0.234, 0.438, and −0.101, respectively); (2) each factor of mental health was significantly positively correlated with psychoticism and neuroticism; only the factor of interpersonal sensitivity was significantly negatively correlated with extraversion; (3) subject type has a significant moderating effect on the relationship between mental health and neuroticism and extraversion, but has no significant moderating effect on the relationship between mental health and psychoticism; (4) publication type does not play a moderating role in the relationship between mental health and the three personality dimensions. This meta-analysis confirmed that personality traits have a significant predictive effect on mental health in Chinese people. However, the relationship between personality and mental health varied considerably across dimensions and groups.

## 1. Introduction

Since the 1980s, the relationship between personality traits and mental health has been studied in Chinese people, and the results have shown that there are significant correlations between them. For example, Zhao et al. found that mental health was correlated with all dimensions of personality, but the highest correlation was found with neuroticism [1]. Chen found that the combination of stressors and personality traits together influenced the mental health of Chinese people [2].

A number of surveys in recent years have produced consistent results: Chinese mental health scores (SCL-90) have a significant positive correlation with psychoticism and neuroticism and a significant negative correlation with extraversion (the measurement tools used in the literature included in the meta-analysis and cited in this study were the SCL-90 and the EPQ; for a detailed description of them, see Section 2.1). For example, Wang and Li surveyed university teaching administrators and found a significant positive correlation between total mental health and psychoticism (r = 0.318), a significant positive correlation with neuroticism (r = 0.553), and a significant negative correlation with extraversion (r = −0.203) [3]. A positive correlation between each factor of mental health and psychoticism and neuroticism and a negative correlation with extraversion were also found by Hu, Wang, and Li among Chinese undergraduates [4]. Another study found that each factor of mental health in Chinese medical graduates had a positive correlation with psychoticism, with correlation coefficients ranging from 0.042 to 0.353, a positive correlation with neuroticism, with correlation coefficients ranging from 0.345 to 0.526, and a negative correlation with extraversion, with correlation coefficients ranging from −0.065 to −0.336 [5]. Lu found in the survey of medical staff that there was a positive correlation between each factor of mental health and psychoticism, with correlation coefficients ranging from 0.175 to 0.330, a positive correlation with neuroticism, with correlation coefficients ranging from 0.451 to 0.463, and a negative correlation with extraversion, with correlation coefficients ranging from −0.087 to −0.253 [6]. Kong found that Chinese nurses’ psychoticism was negatively correlated with the obsessive-compulsive factor and positively correlated with each of the other factors, neuroticism was positively correlated with each factor of mental health, and extraversion was negatively correlated with the interpersonal sensitivity factor and positively correlated with the other factors [7].

In summary, previous studies have shown an association between the dimensions of personality traits and mental health among Chinese people. However, there are significant differences in the results. To explore the reasons for these differences, to further clarify the relationship between mental health and personality, and to resolve the controversies in this area, this study uses a meta-analytic approach to examine the relationship between personality traits and mental health in Chinese people from a macro perspective. The aim is to draw broad and more general conclusions.

## 2. Materials and Methods

### 2.1. Literature Search and Screening

Literature on mental health and personality traits in Chinese people was searched in the three largest Chinese academic databases: CNKI, VIP, and WANFANG. Due to the large amount of relevant literature, the search years for this study were set from 2014 to 2019. A joint keyword search was conducted on mental health and personality. The search terms for mental health were: mental health, SCL-90, and Symptom Self-Rating Scale. The search terms for personality traits were: personality, personality traits, personality characteristics, and EPQ. Literature was included using the following criteria:

(1) Both the Symptom Self-Rating Scale (SCL-90) and the Eysenck Personality Questionnaire (EPQ), revised by Gong Yaoxian, were used in the literature.

SCL-90 (Symptom Checklist 90): Developed by L.R. Derogatis and collaborator, there are a total of 90 items comprising 9 factors: somatization, obsessive-compulsive, interpersonal sensibility, depression, anxiety, anger-hostility, phobic-anxiety, paranoid ideation, and psychoticism. There were also 7 items that were not categorized into any factor and were treated as additional items in order to calculate the total score. The scale is widely used in the field of mental health.

EPQ (Eysenck Personality Questionnaire): A self-report scale developed by the British Psychologist H. J. Eysenck, consisting of four subscales: extraversion scale (E), neuroticism scale (N), psychoticism scale (P), and validity scale (L, or Lying). Extraversion is the directionality and arousal of mental activity, ranging from absolutely introverted to absolutely extraverted. Neuroticism refers to a person’s emotional stability, ranging from extremely stable to extremely unstable. Psychoticism assesses a person’s aggressiveness, creativity, novelty seeking, psychopathy, etc. The scale is widely used in the fields of medicine, justice, and education and is suitable for testing various groups of people;

(2) Complete data were available in the literature: data required for the study were reported, including sample size, Pearson’s correlation coefficient r or other statistical values convertible to Pearson’s correlation coefficient (*T*, *F*, *X*^2^, etc.);
(3) The sample size was clear;
(4) The study population was normal people in China.

Criminals, patients with mental disorders, and patients with physical disorders were excluded from this study. Literature using other measurement instruments or simplified versions of the scales was also excluded. The same data published more than once have been counted only once. Literature reviews and conference abstracts were also excluded.

### 2.2. Coding of Literature Characteristics

Literature meeting the above criteria was coded according to the following characteristics: author, sample size, publication year, effect size, subject group (students, medical staff, soldiers, and others), and publication type (core journals, non-core journals, and theses). Core journals are those included in the Chinese Core Journals Catalogue of Peking University Library, CSSCI of Nanjing University, CSCD of the Chinese Academy of Sciences Literature and Information Centre, and others are non-core journals [8]. Effect sizes were extracted as follows: (1) Correlation coefficients between each dimension and total score of mental health and each factor of personality were included in the coding. (2) Each independent sample was coded only once. If there were multiple independent samples in a study, each independent sample was coded separately.

### 2.3. Publication Bias Control

Publication bias is a common systematic error in meta-analysis. Positive results are more likely to be published than negative results, and therefore, there may be errors in the meta-analysis based on the published literature. It is necessary to discuss publication bias in meta-analysis. The common methods used to find publication bias are funnel plot, linear regression, rank correlation, fail-safe number, and trim and filling [9]. In this study, the fail-safe number method proposed by Rosenthal [10] was used. It indicates the minimum number of unpublished studies (especially those with negative results) that would be needed to reverse the results of a meta-analysis when the results are statistically significant. According to Rosenthal, a fail-safe number greater than 5K + 10 (where K is the number of included studies) is considered to control for publication bias. The larger the fail-safe number, the less affected by publication bias and the more reliable the conclusions of the meta-analysis.

### 2.4. Model Selection

There are two statistical models in meta-analysis: the fixed-effects model and the random-effects model. The fixed-effects model assumes that the effect size parameter is an unknown and fixed constant and that the effect size is an unbiased estimate of the true value. The difference between the effect sizes is due to sampling, and if the sample size is increased, the results will be closer to the true values. The random effects model assumes that the samples of each independent study come from different populations and that the difference in the measurement results of each independent study consists of three parts, namely the true value, the systematic error, and the random error. The difference in results is affected not only by random error but also by systematic error. The systematic error comes from the sampling area, measurement tools, etc., and reflects the internal variation of each study. Each study has its own overall effect, and there is a high degree of variability between studies.

The heterogeneity of effect sizes must be tested in the meta-analysis, and the decision about which model to use is based on the results of the test for heterogeneity. If the results show high heterogeneity, this means that the effect sizes are from different populations, and a random effects model should be used. If the heterogeneity is low, which means that the effect sizes are from the same population, the fixed effects model is used [11]. Because the results of random-effects models are more conservative and reliable, random-effects models tend to be used in real-world studies regardless of the level of heterogeneity [9]. The coding of the literature revealed that the results of the studies included in this meta-analysis varied significantly, making the choice of a random effects model more appropriate. In this study, the choice of model was also determined by the *Q* test and the *I*^2^ value; if the *Q* test result is significant or the *I*^2^ value is higher than 75%, it is more appropriate to use a random effects model and vice versa for a fixed effects model.

### 2.5. Data Analysis

Microsoft Office Excel 2010 was used for literature management, coding of literature characteristics, and basic statistical calculations, and Comprehensive Meta-Analysis Version 2.0 software was used for statistical analysis. The correlation coefficient r was used as an effect size indicator in this study. Effect sizes were calculated as follows: (1) r (Pearson’s correlation coefficient) was selected as the initial effect size based on the data reported by the included original studies. Some studies reported t values, which had to be converted to r. (2) Since r values are not ratio data and are not normally distributed, r values need to be converted to Fisher’s Z values before being calculated. (3) Fisher’s Z is summed and then converted to r. According to Cohen, when the effect size is the correlation coefficient, r ≤ 0.1 indicates a small effect size; r = 0.25 indicates a medium effect size; r ≥ 0.4 indicates a large effect size.

## 3. Results

### 3.1. Literature Inclusion

Once the literature search had been completed, the full text of the papers was carefully read. The literature was screened according to the established inclusion and exclusion criteria, and 70 studies with 72 independent samples of 65,133 subjects were obtained. Fifteen papers were published in core journals, 43 papers in non-core journals, and 12 papers in Master’s thesis. The subject groups were soldiers in 18 papers, medical staff in 7 papers, students in 35 papers, and others in 10 papers. Some of the studies reported correlation coefficients for only one or two of these dimensions. The exact sample size and number of subjects are based on the actual analysis (see Supplementary Materials).

### 3.2. Heterogeneity Test

There are different methods to test for heterogeneity. The method used to test for heterogeneity in this study was the Cochrane Q test. The results (see Table 1) show that the effect sizes Q for the relationship between mental health and the three dimensions of personality traits reached a significant level (*p* < 0.001), which indicates that there is heterogeneity between the results of the studies. *I*^2^ indicates that 93.952%, 98.563%, and 98.185% of the variance comes from the true difference between the effect sizes.

### 3.3. Main Effects Test

According to the results of the heterogeneity test, the random effects model should be used in the main effects test. The results are presented in Table 2. There was a significant correlation between total mental health and psychoticism (r = 0.234, *p* < 0.001); a significant correlation with neuroticism (r = 0.438, *p* < 0.001); and a significant correlation with extraversion (r = −0.101, *p* < 0.05).

The results of the main effects test for the relationship between each mental health factor and personality traits are shown in Table 3. There was a significant positive correlation (*p* < 0.001) between each of the mental health factors and psychoticism, with the Hostility factor having the highest correlation with psychoticism (r = 0.305). There was also a significant positive correlation (*p* < 0.001) between each of the mental health factors and neuroticism, with the Depression factor having the highest correlation with neuroticism (r = 0.478). There was only a negative correlation (r = −0.146, *p* < 0.05) between the Interpersonal Sensitivity factor and extraversion, while the correlation between all other factors and extraversion was not significant.

### 3.4. Moderating Effect Test

If the *I*^2^ value in the heterogeneity test is greater than or equal to 50%, the source of heterogeneity should be investigated. Based on the results of the heterogeneity test, this study discussed the reasons for the differences between the results of previous studies. First, it is examined whether publication types (core journals, non-core journals, and theses) have an effect on the effect size of each study. The statistical results (shown in Table 4) indicate that the Q-values between publication types were 1.398, 0.490, and 1.038 for psychoticism, neuroticism, and extraversion, respectively, none of which were significant (*p* = 0.497, 0.783, and 0.595, in that order). This means that there was no effect of different publication types on the relationship between mental health and the three personality traits, i.e., there were no effects of publication type.

Secondly, it was analyzed whether the subject groups (soldiers, others, students, and medical staff) influenced the effect size of each study. The results showed (see Table 5) that (1) for psychoticism, the subject group Q-value was 3.415, which was not significant (*p* = 0.332). This indicates that the subject group effect was not significant for psychoticism. (2) For neuroticism, Q between subject groups was 8.996, which was significant (*p* = 0.029), and for extraversion, Q between subject groups was 15.410, which was significant (*p* = 0.001). This suggests that the subject group effect is significant for neuroticism and extraversion, i.e., the subject group is one of the reasons for the differences between the results of previous studies.

### 3.5. Publication Bias Test

Publication bias tests were performed on the effect sizes of the included studies, and the results are shown in Table 6. The table shows that the fail-safe numbers of the publication bias test for the effect sizes of total mental health and psychoticism, neuroticism, and extraversion were 29,184, 133,498, and 3749, respectively, all of which were much greater than 5K + 10. This suggests that publication bias was effectively controlled.

## 4. Discussion

This meta-analysis aimed to quantitatively integrate studies conducted between 2014 and 2019 that examined the relationship between mental health and personality traits of Chinese individuals based on two measurement tools: SCL-90 and EPQ. The analysis systematically examined the relationship between mental health and three factors of personality traits. Furthermore, it explored the moderating effects of publication type and subject group. By shedding light on the relationship between mental health and personality traits of Chinese individuals, this study contributes to advancing future research and applications in this field. It is important to acknowledge that studies using the SCL-90 measure have relied on subjective judgments from participants about their symptoms rather than diagnosis according to clinical criteria. Therefore, the results primarily reflect general health status or mental health level rather than the presence of mental illness or the extent of mental disorders in epidemiological terms. Notably, none of the studies included in this meta-analysis involved patients with mental illness. From a psychological services perspective, promoting mental health should focus on cultivating positive personality traits to enhance individuals’ quality of life and subjective well-being.

### 4.1. Analysis of Main Effects

The results of the main effects test showed that the correlation coefficient between total mental health and psychoticism was 0.234, which was a significant positive correlation; the correlation coefficient with neuroticism was 0.438, which was a significant positive correlation; and the correlation coefficient with extraversion was −0.101, which was a negative correlation. The higher the scores of psychoticism and neuroticism, the lower the level of mental health; the higher the scores of extraversion, the higher the level of mental health. These results are consistent with the findings of most Chinese researchers on the relationship between total mental health and personality traits: there is a significant moderate positive correlation between mental health and psychoticism, a significant strong positive correlation with neuroticism, and a significant weak negative correlation with extraversion. In a study, Zhao et al. pointed out that mental health had the highest correlation with neuroticism, and people with neurotic tendencies had higher levels of psychological distress [1]. Xie et al. analyzed the relationship between personality factors and mental health in minority college students and showed that neuroticism had the strongest relationship with mental health, and the higher the neuroticism score, the more pronounced the symptoms [12]. Cheng and Yang found that one of the important factors affecting mental health is emotional stability, and people with stable emotions have better mental health, while those with unstable emotions have poor mental health [13]. Some studies found a higher correlation between mental health and neuroticism [14].

There was a significant positive correlation between each factor of mental health and psychoticism, with correlation coefficients ranging from 0.160–0.305, with the highest correlation between the hostility factor and psychoticism (r = 0.305), which is consistent with the findings of previous studies. A study by Lu [15] found that the higher the psychoticism score, the more pronounced the psychological symptoms, and the psychological symptom with the highest correlation was hostility. A study by Zhang [16] also found the highest correlation between hostility and psychoticism. The present study confirmed that psychoticism can predict hostile symptoms. Individuals with high psychoticism were more likely to be lonely, have difficulty adapting to the external environment, and show hostility in their thoughts, behaviors, and emotions.

There was a significant positive correlation between the mental health factors and neuroticism, with correlation coefficients ranging from 0.341–0.478 and a moderate correlation, which is generally consistent with previous findings. In their study, Cong et al. found that neuroticism could significantly predict depressive symptoms [17], and another study found that high neuroticism predisposed to various mental health problems, including depression [18]. In the present study, neuroticism had the most significant effect on depression, with a correlation coefficient of 0.478. This suggests that neuroticism may be a good predictor of depression. High neuroticism and emotional instability tend to trigger depression, and people with high neuroticism tendencies are a priority population for depression prevention.

Eight factors of mental health were not significantly correlated with extraversion (*p* > 0.05), and only the factor of interpersonal sensitivity was significantly and negatively correlated with extraversion (r = −0.146, *p* < 0.05). One study pointed out that among the mental health factors, only interpersonal sensitivity was significantly correlated with extraversion [19]. Wang, Lin, and Li found that only obsessiveness was not significantly correlated with extraversion, while all other factors were significantly correlated with extraversion [20]. The correlation coefficients between mental health factors and extraversion reported in different studies varied considerably.

### 4.2. Analysis of Moderating Effects

The test for the effect of publication type showed that the Q-values were not significant between publication types (*p* > 0.01) for psychoticism, neuroticism, and extraversion. This means that there was no effect of publication type on the effect size between the results of previous studies. When testing for publication type, it was also found that only effect sizes reported by non-core journals were significant for extraversion (r = −0.124, *p* < 0.001). Further examination of the raw data revealed that the direction of the correlation reported by papers published in non-core journals was generally consistent, but the direction of the correlation reported by papers published in core journals and theses differed significantly. The large difference in raw effect sizes may be one of the reasons for the insignificant correlation reported by papers in core journals and theses. This problem can be addressed by increasing the sample size.

The test for the effect of the subject group showed that none of the Q-values between subject groups were significant (*p* > 0.05) for psychoticism, indicating that there was no subject group effect on the relationship between mental health and psychoticism. In the relationship between mental health, neuroticism, and extraversion, the Q-values between subject groups were significant (*p* < 0.05), which implied a significant subject group moderating effect. The test also revealed that the relationship between extraversion and mental health was significant only for the effect sizes reported by the soldier group papers (r = −0.233, *p* < 0.001). Further examination of the original study data revealed significant differences in the correlations reported by the papers on the other three groups, except for the correlation on the soldiers’ group, which was consistent. Specifically, the correlations for the soldier studies were all negative, and the effect sizes for the student and medical staff studies were positive and negative by ratios of 3:1 and 2:1, respectively. The relative consistency of the original study data may be an important reason for the significant effect sizes reported for the soldiers’ group [11].

This study did not confirm that publication type affected the relationship between mental health and personality traits, which differs from previous findings. Subject groups had different effects on the relationship between the three personality dimensions and mental health. One of the reasons for the difference between the results of this moderating effect test and the results of previous studies may be the size of the sample [21].

### 4.3. Limitations and Perspectives

Firstly, although relevant studies were included as far as possible, it is possible that not all relevant studies were fully included for a number of reasons. For example, some keywords were not included in the search because they did not match the search terms defined for this study. All data in the included studies were published, and data from unpublished studies were not included. For these shortcomings, future studies can collect relevant studies from different perspectives, for example, by widening the search scope to collect as many relevant studies as possible, which could reduce methodological limitations. Secondly, the search year was limited to the last five years. Due to time constraints and the volume of literature, this study could not continue the analysis of studies for longer years. Subsequent researchers could include more studies and increase the sample size by widening the range of search years. Finally, only publication type and subject group were selected as moderating variables. Other moderating variables, such as gender ratio of subjects, age of subjects, age of publication, measurement tools, and cultural background, could be selected in future studies to analyze the effects of study characteristics.

## 5. Conclusions

(1) Total mental health was significantly and positively correlated with psychoticism and neuroticism. It was significantly and negatively correlated with extraversion, but the correlation was weak. (2) Each mental health factor was significantly and positively correlated with psychoticism and neuroticism; only the interpersonal sensitivity factor was significantly and negatively correlated with extraversion. (3) Publication type did not play a moderating role in the relationship between mental health and the three personality traits. (4) The moderating effect of the subject group was not significant in the relationship between mental health and psychoticism but was significant in the relationship between mental health and neuroticism and extraversion.

## Figures and Tables

**Table 1:** Heterogeneity test of effect sizes for the relationship between mental health and personality traits.

Personality Traits	Independent Samples	*Q*	*df*	*p*	*I* ^2^
psychoticism	56	909.372	55	0.000	93.952
neuroticism	64	4383.707	63	0.000	98.563
extraversion	60	3251.404	59	0.000	98.185

**Table 2:** Random Effect Model Analysis Results of the Relationship between Mental Health and Personality Traits.

Personality Traits	Independent Samples	*N*	Effect Sizes and 95% Confidence Interval			Two-Sided Test	
			Point Estimation	Lower Limits	Upper Limits	*Z*	*p*
psychoticism	56	50,738	0.234	0.198	0.270	12.174	0.000
neuroticism	64	56,436	0.438	0.379	0.494	12.909	0.000
extraversion	60	52,631	−0.101	−0.166	−0.035	−3.008	0.003

**Table 3:** Random Effect Model Analysis Results of the Relationship between Mental Health Factors and Personality Traits.

Mental Health Factors	Psychoticism			Neuroticism			Extraversion		
	*r*	*Z*	*p*	*r*	*Z*	*p*	*r*	*Z*	*p*
Somatization	0.224	11.544	0.000	0.371	8.193	0.000	−0.080	−1.361	0.174
Obsessive-compulsive	0.186	9.662	0.000	0.394	6.946	0.000	−0.086	−1.337	0.181
Interpersonal sensibility	0.240	12.828	0.000	0.388	5.744	0.000	−0.146	−2.089	0.037
Depression	0.274	11.970	0.000	0.478	7.362	0.000	−0.133	−1.685	0.092
Anxiety	0.226	11.989	0.000	0.444	7.391	0.000	−0.027	−0.364	0.716
Anger-hostility	0.305	12.634	0.000	0.415	8.467	0.000	−0.080	−1.332	0.183
Phobic-anxiety	0.160	9.684	0.000	0.341	6.253	0.000	−0.095	−1.665	0.096
Paranoid ideation	0.295	15.324	0.000	0.376	7.903	0.000	−0.061	−1.161	0.245
Psychoticism	0.258	12.916	0.000	0.387	6.750	0.000	−0.072	−1.141	0.254

**Table 4:** The moderating effect of publication types on the relationship between mental health and personality traits.

Personality Traits	Heterogeneity Test			Publication Type	*K*	*N*	Effect Sizes and 95% Confidence Interval			Two-Sided Test	
	*Q*	*df*	*p*				Point Estimation	Lower Limits	Upper Limits	*Z*	*p*
psychoticism	1.398	2	0.497	core	7	5336	0.160	0.015	0.298	2.166	0.030
				non-core	38	18,437	0.249	0.197	0.299	9.205	0.000
				thesis	11	26,965	0.231	0.164	0.295	6.634	0.000
neuroticism	0.490	2	0.783	core	10	5966	0.472	0.374	0.559	8.422	0.000
				non-core	42	21,559	0.434	0.350	0.512	9.106	0.000
				thesis	12	28,911	0.423	0.284	0.544	5.549	0.000
extraversion	1.038	2	0.595	core	9	5679	−0.068	−0.202	0.068	−0.983	0.326
				non-core	40	19,987	−0.124	−0.178	−0.069	−4.419	0.000
				thesis	11	26,965	−0.051	−0.226	0.128	−0.554	0.579

**Table 5:** The moderating effect of the subject group on the relationship between mental health and personality traits.

Personality Traits	Heterogeneity Test			Subject Group	*K*	*N*	Effect Sizes and 95% Confidence Interval			Two-Sided Test	
	*Q*	*df*	*p*				Point Estimation	Lower Limits	Upper Limits	*Z*	*p*
psychoticism	3.415	3	0.332	soldiers	17	6920	0.290	0.178	0.395	4.924	0.000
				others *	7	2474	0.243	0.195	0.290	9.584	0.000
				students	27	40,350	0.205	0.163	0.247	9.280	0.000
				medical	5	99	0.188	0.098	0.274	4.062	0.000
neuroticism	8.996	3	0.029	soldiers	17	6920	0.520	0.447	0.587	11.804	0.000
				others	9	2796	0.471	0.405	0.533	12.258	0.000
				students	32	45,646	0.360	0.266	0.448	7.051	0.000
				medical	6	1074	0.559	0.375	0.700	5.228	0.000
extraversion	15.410	3	0.001	soldiers	17	6920	−0.233	−0.298	−0.166	−6.669	0.000
				others	8	2268	−0.065	−0.152	0.023	−1.455	0.146
				students	29	42,300	−0.060	−0.156	0.038	−1.194	0.232
				medical	6	1143	0.052	−0.180	0.280	0.438	0.661

* Others include normal populations such as ship crews, health inspectors, older people, other young people, parents, etc.

**Table 6:** Results of publication bias test for the relationship between total mental health and personality traits.

Personality Traits	Model	Independent Sample	Fail-Safe Number
psychoticism	random effect model	56	29,184
neuroticism		64	133,498
extraversion		60	3749

## Data Availability

Data are available from the corresponding author on reasonable request.

## Supplementary Materials

The following supporting information can be downloaded at: https://www.mdpi.com/article/10.3390/bs13080683/s1. References [22,23,24,25,26,27,28,29,30,31,32,33,34,35,36,37,38,39,40,41,42,43,44,45,46,47,48,49,50,51,52,53,54,55,56,57,58,59,60,61,62,63,64,65,66,67,68,69,70,71,72,73,74,75,76,77,78,79,80] are cited in the Supplementary Materials, which indicate studies included in the meta-analysis.

## References

[1] Zhao J.P., Shen Q.J., Zheng Y.P. (1987). A study on the mental health level and influencing factors of junior medical students. Chin. Ment. Health J..

[2] Chen S.J. (1988). Mental Health and Its Causes among University Students. Chin. J. Appl. Psychol..

[3] Wang C.F., Li J.H. (2017). An investigation and analysis of the mental health status of teaching administrators in Colleges and Universities. Chin. Rural Health Serv. Adm..

[4] Hu Y.Q., Wang B.R., LI Z.H. (2015). Relevant Research on Mental Health, Subjective Well-being and Personality of Post-90s Undergraduates. Univ. Educ. Sci..

[5] Mi Z.H., Chen H.J., Li H.M. (2016). Mental Health and Personality Characteristics of Medical Postgraduates. J. Qingdao Univ..

[6] Lu J. (2015). Investigation and analysis of mental health of medical staff in Jiangyin City. Sichuan Ment. Health.

[7] Kong X. (2015). Analysis on the relationship between mental health and personality characteristics among nurses in a certain tertiary a hospital in Beijing. Chin. J. Health Educ..

[8] Liao Y.G. (2014). Relationship between Coping Style and Mental Health: A Meta-analysis. Chin. J. Clin. Psychol..

[9] Zheng M.H. (2013). Meta-Analysis Software Application and Case Analysis.

[10] Rosenthal R. (1979). The file drawer problem and tolerance for null results. Psychol. Bull..

[11] Ye Z.J. (2010). Meta-Analysis of Researches on the Relationship between Burnout and Mental Health in China. Master’s Thesis.

[12] Xie Y.L., Zhang Y.K., Zhao J.P. (1996). An analysis of the relationship between personality factors and mental health of Ethnic Minorities College Students. Chin. J. Clin. Psychol..

[13] Cheng S.Z., Yang M. (2006). The Investigation of 221 College Students’ Personality Characteristics and Mental Health Status. Mod. Prev. Med..

[14] Zhang Y.P., Zhang Q., Zhao W.B., Zhang N.N., Wang Y.J., An Q. (2015). 564 Basic Unit Officers and Soldiers ‘Mental Health and Personality. China J. Health Psychol..

[15] Lu W.W. (2017). Mental Health Status and Influential Factors of Freshmen in a Medical University in Hebei Province. Master’s Thesis.

[16] Zhang L. (2015). Research on Mental Health and Group Psychological Training in Ethnic Minority Students of Vocational Medical School. Master’s Thesis.

[17] Cong X.Y., Wu Y.Q., He D.J., Li Y., Wang H.F. (2017). Mediating effects of coping styles on neuroticism personality and depression in female nursing college students. Nurs. Integr. Tradit. Chin. West. Med..

[18] Chen L., Zhang F.C., Yin H., Zhang Z.M., Tao Y.C., Xu Y., Wang L., Feng Y. (2018). Mediating effects of neurotic personality between parental rearing patterns and depressive symptom in middle-school students. Chin. J. Behav. Med. Brain Sci..

[19] Li K., Jiang X., Zhao W.L., Wu Z.X., Qiao K. (2016). Investigation on relationship between mental health and personality traits of students left in rural primary school in Gansu province. Bull. Dis. Control Prev..

[20] Wang Y., Lin H.W., Li Y.P. (2014). A study on the relationship between personality characteristics and mental health of Ethnic Minorities College Students in Western Guangxi. J. Campus Life Ment. Health.

[21] Zeng Y. (2008). Meta-Analysis of Researches on the Relationship between Anxiety and Students’ Attainment in the Past Decade in China. Master’s Thesis.

[22] An X., Jia S.L. (2016). The Relationship between University Freshmen’s Mental Health and Parental Rearing Pattern: The Mediating Role of Personality. Contemp. Educ. Cult..

[23] Bao Y.S., Zhu F.W., Cui N., Hu Y. (2018). Moderating effect of conflict resolution on relationship between personalitytraits and interpersonal sensitivity in college students. Chin. Ment. Health J..

[24] Cai Z., Luo X. (2014). The Relationship Between Mental Health and Personality of Freshmen: The Intermediary of Psychological Adaptability. Health Med. Res. Pract..

[25] Cao L. (2010). Psychological Health and Its Related Factors of Employees in a Foreign Enterprise of Suzhou. Occup. Health.

[26] Zeng M.H., Wu J., Zhang Z.T. (2014). A survey of the personality characteristics and mental health of community nurses. Health Vocat. Educ..

[27] Cheng M.Y., Li S.R., Liu X.T., Liu X.Q. (2017). The Relationship Between Mental Health and Personality Characteristics Coping Styles of Empty-nest Elderly. China J. Health Psychol..

[28] Chen Y.Y., Wang M.X., Luan X.R., Liu C.L., Zheng X. (2016). Mental health research for new nurse during the standardized training period. Chin. J. Ind. Hyg. Occup. Dis..

[29] Du G., Weng J. (2016). A longitudinal study on the mental health of the students in Japan and its influencing factors—Taking a vocational and Technical College in Zhejiang as an example. Diet Health.

[30] Feng M., Qi X.Q., Chen L.S., Jiang C., Pu Z.H. A controlled study on changes the psychological status and cognitive function of the parents who have lost their only child. Proceedings of the Annual Academic Meeting of the Psychosomatic Medicine Branch of Zhejiang Medical Association and the Annual Academic Meeting of the Judicial Psychiatry Group of the Psychiatric Branch.

[31] Feng Q., Zhang Q., Zhao W.B., An Q., Zhang N.N. (2018). Investigation on recruits’ personality characteristics status in a military force. Occup. Health.

[32] Guo L.M., Xiao F.M., Jiang Z.M., Lü Z.H., Zhang S.L. (2014). Relationship between Mental Health and Personality Traits in Parents of Children with Cerebral Palsy. Chin. J. Rehabil. Theory Pract..

[33] He C. (2015). The Relationship between Freshmen’s Emotional Stability, Mental Adaptability and Mental Health. Master’s Thesis.

[34] He E.M. (2014). Gender Difference of University Students in Metal Health Personality and Sense of Inferiority. Educ. Teach. Res..

[35] Hu G.T., Feng Z.Z., Wang G.W., Song H., Huang Y., Lu D. (2014). The influence of comprehensive psychological behavior training on psychological stress and health of new recruits in training periods and its interrelation. Chin. J. Behav. Med. Brain Sci..

[36] Hu G.T., Song H., Wang G.W., Feng Z.Z., He Y., Huang Y., Lu D. (2015). Effects of exposed rapidly to high altitude on psychological stress and resilience of automobile soldiers. Med. J. Natl. Defending Southwest China.

[37] Hu G.T., Song H., Wang G.W., Huang Y., Chen X.B., He Y. (2015). A study on psychological stress and its influencing factors of recruits during training. Chongqing Med..

[38] Hu G.T., Wang G.W., Feng Z.Z., Huang Y., He Y., Li Z.H. (2015). The Effect of Comprehensive Psychological Behavior on Mental Elasticity, Self-Consistency and Psychological Stress of automotive Soldiers in Plateau. J. Prev. Med. Chin. People’s Lib. Army.

[39] Huang Y.J. (2016). New Recruits Mental Maladjustment’s Early Prevention and Intervention Evaluation Research. Master’s Thesis.

[40] Jia H.H., Hu G.X., Liu Y., Ye Q.F., Zhu H.M. (2018). A study on the relationship between personality characteristics and mental health of rural left behind women in a county of Hebei Province. J. Clin. Nurs. Pract..

[41] Li C., Yang G.Y., Zhang L., Zhao M.X., Yang X.J., Yu Y., Wang L.F. (2015). Characteristics of mental health in students from Pu’er Medical School, Yunnan Province and its relationship with personality, coping style and trait anxiety. J. Third Mil. Med. Univ..

[42] Li C. (2015). Study on the Mental Health of Medical School Students in Yunnan Border Areas and Education Intervention for Them. Master’s Thesis.

[43] Li K., Wang C., Zhao W.L., Jiang X., Wu Z.X., Pan W.M., Xue L., Qiao K. (2016). The Relationship between Mental Health and Personality Traits of Parents—Absent Students in Junior Middle School of Gansu Province Rural. China J. Health Psychol..

[44] Li W., Peng L.J. (2016). A study on the correlation between mental health level and personality characteristics of recruits. People’s Mil. Surg..

[45] Li Y.F. (2016). Research on Hebei Medical College Freshmen’s Personality Characteristics and Influential Factors. Master’s Thesis.

[46] Lin H.Y. (2015). The relationship between personality, adaptability and mental health of post-90s recruits. J. Chifeng Univ..

[47] Liu L., Sun X.J., Hou X.M., Wang A.Q. (2015). A study on the relationship between stress—Related factors and mental health of family members of the only child with mental disease. J. Qilu Nurs..

[48] Liu M.H., Yu X.Q., Qin L., Jiang G.Q., Yang Y.J., Wei B., Chen J.S. (2017). A related study of psychological status and adverse life events, personality characteristics in adolescents of rural-urban areas. J. Psychiatry.

[49] Liu S.T., Zhang X.J., Lu S., Gao H.B., Liu H.Y., Ma H.M. (2019). Correlation analysis of mental health status and personality characteristics of only-child students in middle school. China J. Health Psychol..

[50] Liu X.Q., Zheng D.W. (2015). Mental health and influencing factors of the rural elderly in Weifang. Chin. J. Gerontol..

[51] Lu S., Xu P., Liu C.L., Chi Q., Xu J.Y., Zhang S., Zhang Y. (2015). A survey of the mental health of officers and soldiers in a Navy Department. J. Chin. Physician.

[52] Lu C.F., Tian Q.X., Li Y.F., Qiao J.H. (2016). Investigation on personality traits and mental health status of head nurses in general hospitals in Shandong province. J. Qilu Nurs..

[53] Ma J.H., Zhang Y.T. (2016). Mediating Effect of the Elderly Social Support between Introversion-extroversion and Mental Health. J. Xinyang Norm. Univ..

[54] Qi L.Y. (2014). An analysis of the personality and mental health of College Students. East West South North.

[55] Qin L. (2015). On the relationships among subjective well-being, personality traits and mental health status of nursing students. Master’s Thesis.

[56] Qiu W.Q. (2018). Psychological Health status and Intervention Study of Recruits in Field Army. Master’s Thesis.

[57] Tian B.W., Huang C.P., Shi C.Y., Zhu J.S., Su Y.N., Qu G.X. (2015). A survey of mental health status and personality characteristics of nurses in our city. Heilongjiang Med. Pharm..

[58] Wang F., Qiao Z.X., Yang X.X., Guo W., Chen M.Q., Wang W.B., Gong P.F., Zhang J., Yang Y.J. (2015). Status and influencing factors of mental health among health supervisors. Chin. J. Public Health.

[59] Wang J., Xu Z.X., Wang T. (2015). Relation of interpersonal sensitivity to personality traits and emotion in air-force soldiers and officers in special environment. Chin. Ment. Health J..

[60] Wang J.L., Yin C. (2014). Analysis of mental health status and influencing factors of marine graduates. Med. J. Commun..

[61] Wang L. (2015). Research on the Relationship between Life Events Personality Traits and Mental Health of University Students-Based on the survey of the students from a university of technology in Guangxi. J. Guilin Norm. Coll..

[62] Wang S.N., Wang X., Zhang H. (2016). A study on the influencing factors of psychological status of nurses in psychiatric hospital. China Health Care Nutr..

[63] Wang T. (2018). The Study of the Relationship between Personality Characteristics and Mental Health of Medical College Freshmen. Master’s Thesis.

[64] Wu Y.H. (2014). Study on the Psychological Health and Education Intervention of Postgraduates in Military University. Master’s Thesis.

[65] Wu Z.Y., Fu X.L., Zhu Y., Wang L.J. (2014). Studies on the correlation between psychological stress status and the personality of the soldiers participating competition in military skills. Pract. J. Med. Pharm..

[66] Xin H.M., Ma J. (2018). A study on the relationship between personality characteristics and mental health of Medical Freshmen. Inn. Mong. Educ..

[67] Xiong H.X., Liu X.Y., Wang X.Q., Tu J.J., Jiang D.D., Zhang J. (2016). Effect of Negative Life Events on Female Students Mental Health: Moderated Mediating Effect. J. Nanchang Norm. Univ..

[68] Xiong J., Su L.N. (2017). The investigation and analysis of the mental health and personality characteristics of the police freshmen. J. Campus Life Ment. Health.

[69] Yan X., Wu J., Ma W.T. (2018). Sleep quality, personality and mental health of soldiers stationed on plateau. Occup. Health.

[70] Yang K., Zhang H. (2016). The influence of left behind junior high school students’ perception of class environment and personality characteristics on mental health. Teach. Adm..

[71] Yang Y.M., Zheng A.M., Chen Y., Li Z., Zhu J., Yang H., Huang Y.H., Zhang X.J., Yang S.C. (2017). Psychological state of officers and soldiers of an armed police force, 2015. Pract. Prev. Med..

[72] Ye M., Tang H.Y., Chen J., Qiu J.M., Chen H.M. (2017). A study on the correlation between the mental health level and personality characteristics of officers and soldiers in field training. People’s Mil. Surg..

[73] Yu H.Z., Yu H.B., Wang Y.X. (2017). Analysis of mental health status and its influencing factors of navy crews during long-term sailing. Acad. J. Second Mil. Med. Univ..

[74] Zhang D.D. (2014). A Study on the Relationship between Personality Characteristics, Coping Styles and Mental Health of New Students in Fire Command School. Master’s Thesis.

[75] Zhang L.H., Han W., Li Y.Q. (2016). The investigation and analysis of the personality and psychological status of the soldiers of a security force in Beijing. Contemp. Med..

[76] Zhang Q., Yin X., Xiao J.C. (2015). Dynamic Analysis on Mental Health Status of Doctoral Students in Hubei and Its Influencing Factors. Psychol. Tech. Appl..

[77] Zhang Z.Q., Gao W.J., Wang J.L. (2014). Correlation between mood stability and mental health of medical students in military academies. J. Logist. Univ. PAPF (Med. Sci.).

[78] Zheng L., Hu T.T., Guo P.F., Zhao H.L. (2014). Social support and subjective well-being: Meta-analysis. Sci. Soc. Psychol..

[79] Zhou Y. (2014). Research on Mental Health Level and Developmental Characteristics of College Students in Chongqing. Master’s Thesis.

[80] Zhu H.Q., Liang L.J., Huang M. (2015). A study on the relationship between mental health and personality characteristics of Medical College Students. J. Campus Life Ment. Health.

