# Supplementary material for: Chinese Personality Traits and Mental Health: A Meta-Analysis
Source: Behav Sci (Basel). 2023 Aug 16;13(8):683. doi: 10.3390/bs13080683 (PMC10451489; doi:10.3390/bs13080683)
Supplement: Supplementary file 1 [file behavsci-13-00683-s001.zip › behavsci-2504607-supplementary.pdf]

Supplementary Materials: References

22. An, X.; Jia, S.L. The Relationship between University Freshmen's Mental Health and Parental Rearing Pattern: The Mediating Role of Personality. *Contemp. Educ. Cult.* **2016**, *8*, 74–79. https://doi.org/10.13749/j.cnki.cn62-1202/g4.2016.06.013.
23. Bao, Y.S.; Zhu, F.W.; Cui, N.; Hu, Y. Moderating effect of conflict resolution on relationship between personalitytraits and interpersonal sensitivity in college students. *Chin. Ment. Health J.* **2018**, *32*, 521–525. https://doi.org/10.3969/j.issn.1000-6729.2018.06.013.
24. Cai, Z.; Luo, X. The Relationship Between Mental Health and Personality of Freshmen: The Intermediary of Psychological Adaptability. *Health Med. Res. Pract.* **2014**, *11*, 17–20.
25. Cao, L. Psychological Health and Its Related Factors of Employees in a Foreign Enterprise of Suzhou. *Occup. Health* **2010**, *32*, 121–122. https://doi.org/10.13329/j.cnki.zyyjk.2010.03.063.
26. Zeng, M.H.; Wu, J.; Zhang, Z.T. A survey of the personality characteristics and mental health of community nurses. *Health Vocat. Educ.* **2014**, *32*, 121–122.
27. Cheng, M.Y.; Li, S.R.; Liu, X.T.; Liu, X.Q. The Relationship Between Mental Health and Personality Characteristics Coping Styles of Empty-nest Elderly. *China J. Health Psychol.* **2017**, *25*, 606–609. https://doi.org/10.13342/j.cnki.cjhp.2017.04.033.
28. Chen, Y.Y.; Wang, M.X.; Luan, X.R.; Liu, C.L.; Zheng, X. Mental health research for new nurse during the standardized training period. *Chin. J. Ind. Hyg. Occup. Dis.* **2016**, *34*, 498–501.
29. Du, G.; Weng, J. A longitudinal study on the mental health of the students in Japan and its influencing factors—Taking a vocational and Technical College in Zhejiang as an example. *Diet Health* **2016**, *3*, 215–217.
30. Feng, M.; Qi, X.Q.; Chen, L.S.; Jiang, C.; Pu, Z.H. A controlled study on changes the psychological status and cognitive function of the parents who have lost their only child. In Proceedings of the Annual Academic Meeting of the Psychosomatic Medicine Branch of Zhejiang Medical Association and the Annual Academic Meeting of the Judicial Psychiatry Group of the Psychiatric Branch, Wenzhou, China, 2014-05-23.
31. Feng, Q.; Zhang, Q.; Zhao, W.B.; An, Q.; Zhang, N.N. Investigation on recruits' personality characteristics status in a military force. *Occup. Health* **2018**, *34*, 2064–2066. https://doi.org/10.13329/j.cnki.zyyjk.2018.0616.
32. Guo, L.M.; Xiao, F.M.; Jiang, Z.M.; LÜ, Z.H.; Zhang, S.L. Relationship between Mental Health and Personality Traits in Parents of Children with Cerebral Palsy. *Chin. J. Rehabil. Theory Pract.* **2014**, *20*, 1068–1070. https://doi.org/10.3969/j.issn.1006-9771.2014.11.018.
33. He, C. The Relationship between Freshmen's Emotional Stability, Mental Adaptability and Mental Health. Master's Thesis, Zhengzhou University, Zhengzhou, China, 2015.
34. He, E.M. Gender Difference of University Students in Metal Health Personality and Sense of Inferiority. *Educ. Teach. Res.* **2014**, *28*, 44–46.
35. Hu, G.T.; Feng, Z.Z.; Wang, G.W.; Song, H.; Huang, Y.; Lu, D. The influence of comprehensive psychological behavior training on psychological stress and health of new recruits in training periods and its interrelation. *Chin. J. Behav. Med. Brain Sci.* **2014**, *23*, 931–934. d0i:10.3760/cma.j.issn.1674-6554.2014.10.020.
36. Hu, G.T.; Song, H.; Wang, G.W.; Feng, Z.Z.; He, Y.; Huang, Y.; Lu, D. Effects of exposed rapidly to high altitude on psychological stress and resilience of automobile soldiers. *Med. J. Natl. Defending Southwest China* **2015**, *25*, 63–66. https://doi.org/10.3969/j.issn.1004-0188.2015.01.024.
37. Hu, G.T.; Song, H.; Wang, G.W.; Huang, Y.; Chen, X.B.; He, Y. A study on psychological stress and its influencing factors of recruits during training. *Chongqing Med.* **2015**, *44*, 329–331. https://doi.org/10.3969 /i. issn.1671-8348.2015.03.014.
38. Hu, G.T.; Wang, G.W.; Feng, Z.Z.; Huang, Y.; He, Y.; Li, Z.H. The Effect of Comprehensive Psychological Behavior on Mental Elasticity, Self-Consistency and Psychological Stress of automotive Soldiers in Plateau. *J. Prev. Med. Chin. People's Lib. Army* **2015**, *33*, 512–515.
39. Huang, Y.J. New Recruits Mental Maladjustment's Early Prevention and Intervention Evaluation Research. Master's Thesis, Xinxiang Medical University, Xinxiang, China, 2016.
40. Jia, H.H.; Hu, G.X.; Liu, Y.; Ye, Q.F.; Zhu, H.M. A study on the relationship between personality characteristics and mental health of rural left behind women in a county of Hebei Province. *J. Clin. Nurs. Pract.* **2018**, *3*, 161–162.
41. Li, C.; Yang, G.Y.; Zhang, L.; Zhao, M.X.; Yang, X.J.; Yu, Y.; Wang, L.F. Characteristics of mental health in students from Pu'er Medical School, Yunnan Province and its relationship with personality, coping style and trait anxiety. *J. Third Mil. Med. Univ.* **2015**, *37*, 2400–2404.
42. Li, C. Study on the Mental Health of Medical School Students in Yunnan Border Areas and Education Intervention for Them. Master's Thesis, Third Military Medical University (China), Chongqing, China, 2015.
43. Li, K.; Wang, C.; Zhao, W.L.; Jiang, X.; Wu, Z.X.; Pan, W.M.; Xue, L.; Qiao, K. The Relationship between Mental Health and Personality Traits of Parents—Absent Students in Junior Middle School of Gansu Province Rural. *China J. Health Psychol.* **2016**, *24*, 1557–1560. https://doi.org/10.13342/j.cnki.cjhp.2016.10.032.

44. Li, W.; Peng, L.J. A study on the correlation between mental health level and personality characteristics of recruits. *People's Mil. Surg.* **2016**, *59*, 1103–1105.
45. Li, Y.F. Research On Hebei Medical College Freshmen's Personality Characteristics and Influential Factors. Master's Thesis, Hebei Medical University, Hebei, China, 2016.
46. Lin, H.Y. The relationship between personality, adaptability and mental health of post-90s recruits. *J. Chifeng Univ. (Nat. Sci. Ed.)* **2015**, *31*, 173–174. https://doi.org/10.13398/i.cnki.issn1673-260x.2015.14.071.
47. Liu, L.; Sun, X.J.; Hou, X.M.; Wang, A.Q. A study on the relationship between stress—Related factors and mental health of family members of the only child with mental disease. *J. Qilu Nurs.* **2015**, *21*, 11–13. https://doi.org/10.3969 /j.issn.1006-7256.2015.05.006.
48. Liu, M.H.; Yu, X.Q.; Qin, L.; Jiang, G.Q.; Yang, Y.J.; Wei, B.; Chen, J.S. A related study of psychological status and adverse life events, personality characteristics in adolescents of rural-urban areas. *J. Psychiatry* **2017**, *30*, 326–328. https://doi.org/10.3969 /j.issn.2095- 9346.2017.05.002.
49. Liu, S.T.; Zhang, X.J.; Lu, S.; Gao, H.B.; Liu, H.Y.; Ma, H.M. Correlation analysis of mental health status and personality characteristics of only-child students in middle school. *China J. Health Psychol.* **2019**, *27*, 620–622. https://doi.org/10.13342/j.cnki.cjhp.2019.04.036.
50. Liu, X.Q.; Zheng, D.W. Mental health and influencing factors of the rural elderly in Weifang. *Chin. J. Gerontol.* **2015**, *35*, 4339–4341. https://doi.org/10.3969 /j. issn.1005-9202.2015.05.107.
51. Lu, S.; Xu, P.; Liu, C.L.; Chi, Q.; Xu, J.Y.; Zhang, S.; Zhang, Y. A survey of the mental health of officers and soldiers in a Navy Department. *J. Chin. Physician* **2015**, z1, 199–200. https://doi.org/10.3760/j.issn.1008-1372.2015.z1.096.
52. Lu, C.F.; Tian, Q.X.; Li, Y.F.; Qiao, J.H. Investigation on personality traits and mental health status of head nurses in general hospitals in Shandong province. *J. Qilu Nurs.* **2016**, *22*, 19–21. https://doi.org/10.3969/j.issn.1006-7256.2016.03.008.
53. Ma, J.H.; Zhang, Y.T. Mediating Effect of the Elderly Social Support between Introversion-extroversion and Mental Health. *J. Xinyang Norm. Univ.* **2016**, *36*, 31–35.
54. Qi, L.Y. An analysis of the personality and mental health of College Students. *East West South North* **2014**, *10*, 3–4.
55. Qin, L. On the relationships among subjective well-being, personality traits and mental health status of nursing students. Master's Thesis, Wannan Medical College, Wuhu, China, 2015.
56. Qiu, W.Q. Psychological Health status and Intervention Study of Recruits in Field Army. Master's Thesis, Nanchang University, Nanchang, China, 2018.
57. Tian, B.W.; Huang, C.P.; Shi, C.Y.; Zhu, J.S.; Su, Y.N.; Qu, G.X. A survey of mental health status and personality characteristics of nurses in our city. *Heilongjiang Med. Pharm.* **2015**, *38*, 14–16.
58. Wang, F.; Qiao, Z.X.; Yang, X.X.; Guo, W.; Chen, M.Q.; Wang, W.B.; Gong, P.F.; Zhang, J.; Yang, Y.J. Status and influencing factors of mental health among health supervisors. *Chin. J. Public Health* **2015**, *31*, 1409–1411. https://doi.org/10.11847/zgggws2015-31-11-13.
59. Wang, J.; Xu, Z.X.; Wang, T. Relation of interpersonal sensitivity to personality traits and emotion in air-force soldiers and officers in special environment. *Chin. Ment. Health J.* **2015**, *29*, 952–956. https://doi.org/10.3969/j.issn.1000-6729.2015.12.012.
60. Wang, J.L.; Yin, C. Analysis of mental health status and influencing factors of marine graduates. *Med. J. Commun.* **2014**, *28*, 69–74.
61. Wang, L. Research on the Relationship between Life Events Personality Traits and Mental Health of University Students-Based on the survey of the students from a university of technology in Guangxi. *J. Guilin Norm. Coll.* **2015**, *29*, 102–106. https://doi.org/1016020/jcnkicn45-1302/z.2015.04.020.
62. Wang, S.N.; Wang, X.; Zhang, H. A study on the influencing factors of psychological status of nurses in psychiatric hospital. *China Health Care Nutr.* **2016**, *26*, 167.
63. Wang, T. The Study of the Relationship between Personality Characteristics and Mental Health of Medical College Freshmen. Master's Thesis, Hebei Medical University, Hebei, China, 2018.
64. Wu, Y.H. Study on the Psychological Health and Education Intervention of Postgraduates in Military University. Master's Thesis, Third Military Medical University (China), Chongqing, China, 2014.
65. Wu, Z.Y.; Fu, X.L.; Zhu, Y.; Wang, L.J. Studies on the correlation between psychological stress status and the personality of the soldiers participating competition in military skills. *Pract. J. Med. Pharm.* **2014**, *31*, 868–870. https://doi.org/10.14172/j.cnki.issn1671-4008.2014.10.002.
66. Xin, H.M.; Ma, J. A study on the relationship between personality characteristics and mental health of Medical Freshmen. *Inn. Mong. Educ.* **2018**,04, 92–94.
67.Xiong, H.X.; Liu, X.Y.; Wang, X.Q.; Tu, J.J.; Jiang, D.D.; Zhang, J. Effect of Negative Life Events on Female Students Mental Health: Moderated Mediating Effect. *J. Nanchang Norm. Univ. (Compr.)* **2016**, *37*, 112–118.
68. Xiong, J.; Su, L.N. The investigation and analysis of the mental health and personality characteristics of the police freshmen. *J. Campus Life Ment. Health* **2017**, *15*, 121–122.
69. Yan, X.; Wu, J.; Ma, W.T. Sleep quality, personality and mental health of soldiers stationed on plateau. *Occup. Health* **2018**, *34*, 474–476. https://doi.org/10.13329/j.cnki.zyyjk.2018.0134.

70. Yang, k.; Zhang, H. The influence of left behind junior high school students' perception of class environment and personality characteristics on mental health. *Teach. Adm.* **2016**, *9*, 26–29.
71. Yang, Y.M.; Zheng, A.M.; Chen, Y.; Li, Z.; Zhu, J.; Yang, H.; Huang, Y.H.; Zhang, X.J.; Yang, S.C. Psychological state of officers and soldiers of an armed police force, 2015. *Pract. Prev. Med.* **2017**, *24*, 1179–1184. https://doi.org/10.3969/j.issn.1006-3110.2017.10.008.
72. Ye, M.; Tang, H.Y.; Chen, J.; Qiu, J.M.; Chen, H.M. A study on the correlation between the mental health level and personality characteristics of officers and soldiers in field training. *People's Mil. Surg.* **2017**, *60*, 6–8.
73. Yu, H.Z.; Yu, H.B.; Wang, Y.X. Analysis of mental health status and its influencing factors of navy crews during long-term sailing. *Acad. J. Second Mil. Med. Univ.* **2017**, *38*, 506–510. https://doi.org/10.16781/1.0258-879x.2017.04.0506.
74. Zhang, D.D. A Study on the Relationship between Personality Characteristics, Coping Styles and Mental Health of New Students in Fire Command School. Master's Thesis, Yunnan Normal University, Kunming, China, 2014.
75. Zhang, L.H.; Han, W.; Li, Y.Q. The investigation and analysis of the personality and psychological status of the soldiers of a security force in Beijing. *Contemp. Med.* **2016**, *22*, 3–4. https://doi.org/10.3969 j.iss.1009-4393.2016.31.002.
76. Zhang, Q.; Yin, X.; Xiao, J.C. Dynamic Analysis on Mental Health Status of Doctoral Students in Hubei and Its Influencing Factors. *Psychol. Tech. Appl.* **2015**, *11*, 13–17. https://doi.org/10.16842/j.cnki.issn2095-5588.2015.11.004.
77. Zhang, Z.Q.; Gao, W.J.; Wang, J.L. Correlation between mood stability and mental health of medical students in military academies. *J. Logist. Univ. PAPF (Med. Sci.)* **2014**, *23*, 592–595. https://doi.org/10.3969/iissn2095-3720.2014.07.012.
78. Zheng, L.; Hu, T.T.; Guo, P.F.; Zhao, H.L. Social support and subjective well-being: Meta-analysis. *Sci. Soc. Psychol.* **2014**, *29*, 3–9.
79. Zhou, Y. Research on Mental Health Level and Developmental Characteristics of College Students in Chongqing. Master's Thesis, Southwest University, Chongqing, China, 2014.
80. Zhu, H.Q.; Liang, L.J.; Huang, M. A study on the relationship between mental health and personality characteristics of Medical College Students. *J. Campus Life Ment. Health* **2015**, *13*, 91–93.
